# Topologically trivial graphene enables mid-infrared orbital angular momentum detection toward on-chip integration

**DOI:** 10.1038/s41377-025-01829-7

**Published:** 2025-04-02

**Authors:** Jiayue Han, Jun Wang

**Affiliations:** 1 School of Optoelectronic Science and Engineering, University of Electronic Science and Technology of China, Chengdu, 610054 China; 2 State Key Laboratory of Electronic Thin Films and Integrated Devices, University of Electronic Science and Technology of China, Chengdu, 610054 China

**Keywords:** Optical physics, Optical properties and devices

## Abstract

A mid-infrared orbital angular momentum detector based on multilayer graphene has been successfully developed, overcoming the previous reliance on C_2V_ point group topological Weyl semimetals via the orbital photogalvanic effect. This CMOS-compatible two-dimensional material system is crucial for advancing the large-scale practical application of orbital angular momentum detectors.

## Abstract (web summary)

A mid-infrared orbital angular momentum detector based on multilayer graphene has been successfully developed, overcoming the previous reliance on C_2V_ point group topological Weyl semimetals via the orbital photogalvanic effect.

## Graphical abstract

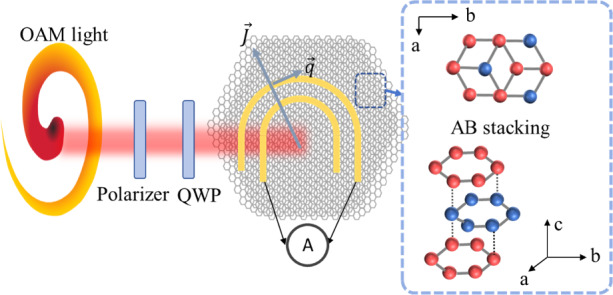

Vortex light can simultaneously carry both spin angular momentum (SAM) and orbital angular momentum (OAM), with the two maintaining an independent relationship^1,2^. This has attracted significant attention because of its potential to enhance applications in optical communication, quantum information processing, optical micromanipulation, and microscopy^3–6^. Traditional optical OAM detection methods are bulky and involve complex fabrication processes, which create barriers for on-chip integration and high-speed applications^5^. To meet the demands of next-generation compact, high-capacity nanophotonic applications, miniaturized, direct on-chip OAM detection has become a key focus for researchers.

Currently, researchers have primarily developed two types of direct OAM detectors: those based on surface plasmon polaritons (SPP)^7,8^ and those based on the orbital photogalvanic effect (OPGE)^9,10^. These two types of detectors significantly differ in response speed, with the latter requiring external circular polarization modulation, whereas the former suffering from relatively low coupling efficiency. OAM detectors based on C_2V_ point group topological Weyl semimetals^11,12^, such as WTe_2_ and TaIrTe_4_, in conjunction with U-shaped electrodes that couple current winding effects, rely on this modulation to obtain the OPGE response component. The response magnitude is mainly determined by the material’s energy dispersion and scattering rate. However, the stability and responsivity of current materials hinder the future development of large-area, high-speed applications for such OAM detectors in focal plane systems.

In a newly published paper in Light: Science & Applications, a direct mid-infrared OAM detector has been proposed on the basis of multilayer graphene and a U-shaped electrode architecture by Dehong Yang and coworkers from the School of Physics, Peking University, and Changchun Institute of Optics, Chinese Academy of Sciences^13^. Compared with WTe2 and TaIrTe4, this device has a stronger OPGE response and a larger signal-to-noise ratio with a responsivity of 24.7 µA/W due to the photothermoelectric (PTE) and photovoltaic (PV) effects at 4 µm.

Instead of using traditional materials that are based on C_2V_ point group (C_2V_ materials is hard to break inversion symmetry under normal incidence), OAM detectors are realized using graphene with high symmetry of the D_6h_ point group (as shown in Fig. 1a). On the basis of the symmetry of multilayer graphene, in a U-shaped electrode device, second-order photoelectric currents in the plane depend on four terms related to normal incidence ((1)–(4)). The collected *J*(1) has a nonzero radial component proportional to the OAM order m, whereas the circular photogalvanic effect (CPGE) background is not collected and the output current is unaffected by circularly polarized modulation (as shown in Fig. 1b). Therefore, in a U-shaped electrode multilayer graphene device, various OAM orders can be effectively distinguished and are proportional to the order. In contrast, in the starfish-type electrode device, for multilayer graphene (Fig. 1c), (1) has no azimuthal component, and (3) is not dependent on the OAM. Therefore, only the CPGE background contributes to the azimuthal response component of (3).

**Fig. 1:**
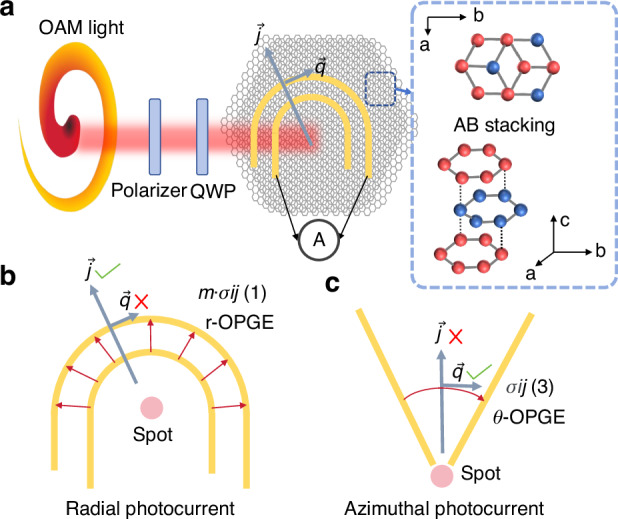
Schematic illustration of dimensionality-enhanced mid-infrared light vortex detection. **a** Schematic diagram of U-shaped electrode multilayer graphene device for direct OAM light detection. **b** Analysis of the radial current in a U-shaped electrode device. **c** Analysis of the azimuthal currents in the starfish electrode device

Their experiments revealed that low scattering rate of multilayer graphene and the reduction in the dimensionality of the linear dispersive energy band are the primary reasons for the increase in the OPGE response. This is because the interlayer coupling in multilayer graphene is very weak and does not affect the intrinsic linear energy dispersion of graphene. In contrast, both WTe_2_ and TaIrTe_4_ have a limited linear dispersion region and a lower Fermi velocity, resulting in a smaller response. Additionally, the low scattering rate originates from differences in crystal quality, whereas graphene is very high quality with a low density of defects, both WTe_2_ and TaIrTe_4_ are unstable under room-temperature atmospheric environment.

Unlike the significant technical barriers associated with large-area topological Weyl semimetals, multilayer graphene offers a wafer-scale solution through chemical vapor deposition and is already compatible with CMOS technology. Furthermore, multilayer graphene demonstrates unique properties in U-shaped electrode systems under normal incidence, where the CPGE component of the photon drag current is suppressed, revealing a forbidden effect for the photoelectric phenomenon. Traditional OPGE-based approaches require simultaneous measurements of photoelectric currents generated by left- and right-handed circularly polarized light to extract the OPGE current, often reducing the accuracy of orbital angular momentum (OAM) detectors. This study fundamentally overcomes these limitations by amplifying response signals, thus enhancing the accuracy and practicality of OAM detection. Additionally, it provides new insights into the material symmetry requirements for OPGE-based OAM detection. The proposed device supports focal plane detector fabrication for diverse applications. Moreover, high-symmetry D_6h_ point group materials, particularly semiconductors like transition-metal dichalcogenides (TMDs), may further advance OAM detection by enabling low-noise, large-area applications.

Recently, Dai et al. from the Center for Optoelectronics and Biophotonics, School of Electrical & Electronic Engineering, Nanyang Technological University, developed an on-chip OAM detector using SPP technology^8^. By integrating 2D optothermal material PdSe_2_ with a parallel aperture column array SPP pattern, they created a spatially unified device. This system determines the phase information of vortex beams based on photoelectric current amplitude and polarity, achieving speeds optimized to tens of microseconds. Unlike conventional focused SPP technologies, which require microscopes and additional designs to separate spatial information, this approach enables direct on-chip OAM information extraction via photo-voltage amplitude and polarity. Furthermore, employing thermoelectric materials extends the detection wavelength range to the mid-infrared region.

Both SPP and OPGE technologies exhibit unique advantages and limitations. OPGE systems feature simple detection structures but suffer from slower speeds due to external circular polarization modulation. Conversely, SPP technologies enable the detection of a broader range of angular momentum states but are hindered by low coupling efficiency and high propagation losses. Recently, both methods have achieved on-chip integration and mid-infrared wavelength extension. Future advancements in OAM focal plane detectors could emerge by integrating the strengths of these direct detection techniques while addressing their limitations. A deeper understanding of material symmetries, physical properties, and synergistic technological combinations is essential. Such developments are expected to facilitate the creation of high-speed, high-sensitivity, and compact OAM detectors, promoted their adoption in industrial and commercial applications.
